# Evaluating the Effectiveness of Childbirth Preparation Courses on Women’s Self-Efficacy among Ultra-Orthodox Jewish Religious Women in Israel

**DOI:** 10.3390/healthcare9070818

**Published:** 2021-06-28

**Authors:** Liat Korn, Gideon Koren, Ayelet Yaakov, Galit Madar, Ayala Blau

**Affiliations:** 1 Department of Health Systems Management, School of Health Sciences, Ariel University, 40700 Ariel, Israel; Ayeletyakov@gmail.com; 2 Adelson School of Medicine, Ariel University, 40700 Ariel, Israel; gidiup_2000@yahoo.com; 3 School of Communication, Ariel University, 40700 Ariel, Israel; galit.stat18@gmail.com; 4 Department of Nursing, School of Health Sciences, Ariel University, 40700 Ariel, Israel; Ayalabl@ariel.ac.il

**Keywords:** childbirth preparation course, primigravid, self-efficacy, ultra-orthodox, birth

## Abstract

**Background:** This study examined the effectiveness of a birth preparation course on coping with childbirth among primigravid ultra-orthodox Jewish women in Israel. **Methods:** In total, 130 ultra-orthodox 25–35-week primigravid women were divided into a study (*n* = 100, participated in birth preparation courses) and a control (*n* = 30, did not participate in the courses) group. A questionnaire was delivered three times: T_1_—before the course/delivery, T_2_—two–three days after delivery, and T_3_—a month after delivery. **Results:** At T_3_, self-efficacy among the study group was higher than in the control group. Differences in self-efficacy were found over time regardless of the group (F_(2,246)_ = 12.83, *p* < 0.001), as a time–group interaction effect (F_(2,246)_ = 10.20, *p* < 0.01). Self-efficacy in the study group (Mean, M = 3.40, Standard deviation, SD = 0.63 at T_1_) dropped to M = 3.06, SD = 0.76 at T_2_ and rose to M = 3.34, SD = 0.64 at T_3_. In the control group, self-efficacy (M = 3.53, SD = 0.56 at T_1_) dropped to M = 3.26, SD = 0.63 at T_2_ and to M = 2.95, SD = 0.76 at T_3_. **Discussion:** The childbirth preparation course was found to be effective in raising self-efficacy among primigravid ultra-orthodox religious women when compared to the control group.

## 1. Introduction

Fear of childbirth has been found to be directly related to fear of pain, lowering mother’s confidence in her natural ability to give birth and leading to the use of painkillers and instrumental births [1]. “Tocophobia” refers to anxiety about childbirth and seeking to give birth by caesarean section even though there is no medical reason for it [2]. While most women are afraid of labor pain, they come to terms with it. They seek to give birth in a supportive, pleasant, respectful environment with staff that knows how to respond accordingly. They need reinforcement in their self-efficacy to give birth naturally, as their mothers and grandmothers did [3]. Natural childbirth has physiological and psychological benefits for women and their families and for health care cost reduction [1].

In the context of childbirth, women with high levels of anxiety tend to opt for an epidural injection as compared to women with low fear levels [4,5]. Self-efficacy was defined by Lowe (1993) as one’s belief of the ability to perform a behavior successfully in a particular context [6]. A review has found that self-efficacy has a positive relationship with physiological and mental health outcomes. High self-efficacy at birth was associated with low-risk pregnancies, fewer caesarean sections, less labor pains and suffering, and low postpartum depression rates [7]. Among 276 pregnant Spanish women, positive associations were found between self-efficacy in childbirth and the use of coping strategies, better positive assessment of the birth experience, and higher satisfaction with childbirth compared to women with lower self-efficacy [8]. Women with low self-efficacy tended to choose a caesarean section instead of natural birth and continued to request a caesarean section in further pregnancies [9]. Self-efficacy at birth was also associated with self-resilience and vigor [4]. According to the theory of self-efficacy [10,11], experience serves as a means of strengthening self-efficacy. In the case of primigravid women, self-efficacy is not gained from previous experience

Childbirth preparation courses aim to help expectant mothers make decisions before and during childbirth, make use of skills they have learned in the course for self-control of labor pain, baby care, breastfeeding, and motherhood skills [12]. Women should feel at the end of this course they have the skills and confidence to take actions that will contribute to successful pregnancy, childbirth, and early parenting [13,14]. On the other hand, childbirth preparation courses are artificial, with the aim of being a substitute for information received by women in the “female network”, and the question remains how effective they are. Courses vary in length depending on economic constraints and structural changes in the health system [15]. In a review of 10 papers from Spain, Sweden, Canada, Australia, Iran, Thailand, and the US, findings regarding these courses’ effectiveness were not uniform [16]. However, in general, childbirth preparation courses were found to have a positive effect on reducing mother’s anxiety and stress. In Israel, a study on primigravid women found that courses reduce stress but do not increase self-efficacy [5].

The goal of the present study was to examine the effectiveness of birth preparation courses in affecting the self-efficacy of Israeli Jewish ultra-orthodox primigravid women. This population is grossly understudied, despite its high fertility rate. The study examined differences in self-efficacy between women who took a birth preparation course and women who did not take a course.

Ultra-orthodox Jews in Israel were about 8%–11% of the Israeli population in 2018, with a high fertility rate—around three times that of non-orthodox Jews. Having a baby is considered a fulfillment of a spiritual purpose. They live in closed neighborhoods with a lifestyle based on Jewish law—the Torah and subsequent writings. They avoid accessing the internet and wear modest clothes. While the men spend their days studying, the women take charge of the house, including income and children’s education [17].

## 2. Methods

### 2.1. Procedure

This was a comparative quantitative study that included a longitudinal follow-up in groups examined at three time points: 1. before the course/before birth during pregnancy (T_1_); 2. two-three days after birth, before participants were released from the maternity department.; 3. two weeks to one month after birth (T_3_). The decision to carry out the third data collection about a month after birth was based on our wish to allow sufficient recovery time from the birth and, on the other hand, not to wait too long, so to avoid forgetting what learned. The study received approval from the Institutional Ethics Committee and administrative approval from the medical center. Six birth preparation instructors delivered the same courses and were informed and trained on the study and its objectives. The courses were held both in the hospital and in cities in the center of Israel: Petah Tikva, Bnei Brak, Elad, and Ashdod. After obtaining written informed consent to participate in the study, questionnaires were distributed to the participating women. Subsequently, at the next point in time at the end of the course, the instructors distributed the questionnaires to the women who met the inclusion criteria at the last session of the course. The women provided telephone contact information. The control group was recruited in women’s health services clinics in the cities of Bnei Brak and Elad during a glucose tolerance test performed at 28–24 weeks of pregnancy. These women were contacted directly after receiving approval from the clinic director. They were explained the purpose of the study and then asked for their consent to participate in the study and were included under the condition of expressing unwillingness to attend the course. In this study, participants were selected to be part of the intervention group from women attending a number of courses delivered to primigravid over several months. The 100-women group representing the experimental group had a strong statistical power. For the control group, 30 subjects were recruited.

Inclusion criteria to the study group included primigravid women who wished to participate in childbirth preparation courses and agreed to participate in the study and join all 4 first meetings in the course. Inclusion criteria to the to control group included primigravid women who did not wish to participate in the childbirth preparation courses and agreed to participate in the study. Exclusion criteria to the study group included women who did not attend the four meetings. About 10% of the participants in each course did not participate in the study, mostly due to fear of answering the pregnancy questions. Specifically, they were afraid to disclose details about their pregnancy without the approval of their rabbi.

### 2.2. Sample

This study aimed to represent the population of Israeli Jewish ultra-orthodox primigravid women. The total sample included 130 ultra-orthodox 25–35-week primigravid women that were divided into a study (*n* = 100, who were participating in six birth preparation courses) and a control (*n* = 30, did not participate) group. The control group socio-demographic characteristics were not significantly different from those of the study group, except for mean age. The sample size was calculated using G* power and based on a power of 0.8 and a medium effect size (d = 0.5) with significance level of 5% as reported before [12,13]. Participants in the study group joined 6 different courses and were easy to access in this framework. The control group participants were much harder to recruit, as they did not join a specific framework and needed to be sure they do not want to participate in a course at the time. Therefore, an allocation ratio of 3:1 was used. The sample size calculated was 100 participants for the study group and 34 for the control group.

### 2.3. Research Tool

Self-reported questionnaires were filled at the clinic or over the phone three times for each participant, before and after delivery: At T_1_—a prenatal questionnaire as well as a pre-course preparation for the intervention group, which provided the basic data for both study and control groups and was designed to assess mother’s self-ability to cope successfully with the birth. At T_2_, and T_3_—a questionnaire after the birth as well as after the childbirth preparation course in the intervention group provided the post data. Subjects were also asked about socio-demographic details and general details about pregnancy and childbirth. Self-efficacy was based on the questionnaire developed by Lowe (1993) “Childbirth Self-Efficacy Inventory” (CBSEI), that assesses a woman’s ability to cope with childbirth [6]. The CBSEI was translated to Hebrew by a Jewish religious translator, culturally adjusted to the ultra-orthodox community and validated in a pilot study with several students.

### 2.4. Variable Description

*Socio-demographic data* included age, marital status, country of birth, year of immigration, education, city of residence, as well as details of pregnancy—gestational age, course of pregnancy (normal/abnormal including details), and participation in a childbirth preparation course. *Physiological data* included gestational age at birth, type of birth, the use of painkillers, type of induction of labor, opening, duration of birth, and breastfeeding. *The dependent variable-* Self-efficacy questionnaire at birth. The questionnaire was developed by Lowe in 1993 [6] and called “Childbirth Self-Efficacy Inventory”; it wis based on Bandura’s (1997) self-efficacy theory. The purpose of the questionnaire is to assess a woman’s ability to cope with childbirth. The assessment is based on a16-item questionnaire with Cronbach’s Alpha Questionnaire Reliability of 0.90 [18]. The questionnaire was given to each mother 3 times. The first time, before birth (OE, Outcome Expectancy), the second time, in the simulation of the birth itself (EE, Efficacy Expectancy), and the third time after birth (CE, Coping ability). The answer evaluation scale is built according to the Likert scale, which ranges from 1 to 5.

In all measurements, the same questions are asked, but the evaluation scale of the answers is different. Outcome Expectancy (OE)—the response evaluation scale in the first measurement is: 1 does not help at all, 5 helps to a very large extent (Cronbach’s Alpha 0.889). Efficacy Expectancy (EE)—the evaluation scale of the answers in the second measurement is: 1 not at all certain, 5 very safe (Cronbach’s Alpha 0.921). Coping ability (CE)—the evaluation scale of the answers in the third measurement is: 1 I did not use at all, 5 I used to a large extent (Cronbach’s Alpha 0.923).

### 2.5. Description of the Intervention—Childbirth Preparation Course

The courses took place in community centers or at the auditorium of the Mayanei HaYeshua Medical Center. Each course was attended by 15 to 20 women. The intervention groups included subjects from seven groups. The course was conducted over 5 sessions and scheduled once a week over a month. Each session lasted about four hours during the evening with a forty-minute break in the middle for questions and refreshments. The four first meetings were delivered by an obstetrician, and the last one was split to two audiences: the men had a lecture from a rabbi, and the women had a lecture from a spiritual religious leader. The content of the first meeting included the physiology of pregnancy and childbirth and used presentations with pictures and movies that reflected and explained the side effects of pregnancy, pregnancy tests needed to be taken during the nine months of pregnancy, tests performed during each month of pregnancy, and so on. Ultra-orthodox women mostly avoid pregnancy tests such as nuchal transparency and amniotic fluid recommended by their rabi; therefore, the explanation usually focuses on the first and second screening tests and on the glucose tolerance test. The second meeting dealt mostly with the three stages of birth. Presentations integrated videos that explained the birth stages, breathing techniques, relaxation exercises, physio ball, using painkillers, and so on. This session is the most important for the sense of efficiency. The third meeting was about getting better after birth, physiologically and psychologically. It dealt with moods, understanding the new relationship between mother and baby and father, the importance of the father in rearing the baby, and how to seek help when needed. The fourth meeting started with a 40-min video that helped to understand the physiology of the breast, what affects breastfeeding, how to breastfeed, practicing while sitting. It finished with scheduling the fifth meeting with the spouses. At the last meeting, each woman was accompanied by her mother and spouse, but then, separately, men talked with the rabbi mostly regarding the importance of religious laws on pregnancy and childbirth. Mothers and daughters had a talk about mind and spirituality in childbirth. The mother was considered as the main caregiver, and the issues were addressed mostly to her. All women participating in the study attended all intervention meetings from 7 courses. As suggested by Lowe [6], these intervention actions were proposed to enlarge women’s belief of their ability to successfully perform an appropriate behavior in the context of childbirth.

### 2.6. Data Analysis

**Figure 1:**
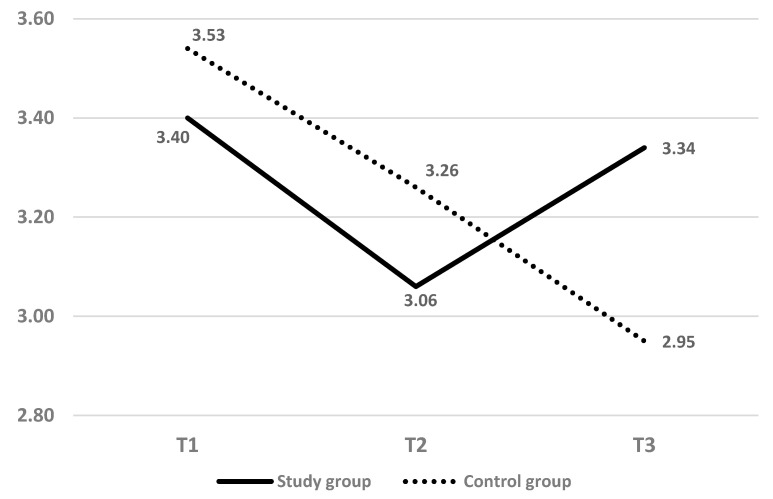
Mean score of childbirth self-efficacy at T_1_–T_3_ (weighted of age).

Data were analyzed using IBM SPSS Statistics 25. Table 1 presents demographic and birth data using cross-tabulation frequencies and Pearson chi-square test for differences between independent groups. Table 1 also shows means, standard deviations for differences between independent groups using t-test according to the nature of the variables for the unweighted sample. As the ages of the study and control groups were significantly different, a weight procedure was implemented before data analysis. Figure 1 shows the mean scores of the study and the control groups at T_1_–T_3_ (weighted for age). Table 2 shows the means and standard deviations by independent t-test analysis and repeated measures for the interaction effect between and within subjects. Further analyses were based on Bonferroni correction to examine the different measurements.

Table 2 further elaborates on the main and interaction effects. 

## 3. Results

The socio-demographic characteristics of the study participants by groups, as well as pregnancy and birth variables, are presented in Table 1.

The total sample’s mean age was 24.0 years (SD = 4.9). Mean age was the only significant difference between the study and control groups (for the study group, M = 23.4, SD = 4.3; for the control group, M = 26.1, SD = 6.1). The vast majority of the participants were married (99.2%) and born in Israel (93.8%), and half of them had an academic or professional education (50.4%). The respondents answered the first questionnaire at a mean age of 33 weeks of gestation (SD = 4.4) and gave birth at week 39.6 (SD = 1.3); most had a natural birth (79.1%), 8.5% had a caesarean section, and 12.3% had an instrumental birth. Most participants stated that the pregnancy was normal (87.7%), chose to take a painkiller or an epidural (89.2%), and later reported that they were breastfeeding (93.8%). While not significantly different, the natural birth method was more frequent in the control group (86.7%) than in the study group (76.8%). Healthy pregnancy (89.0% vs. 83.3%) and usage of pain relief (91.0% vs. 83.3%) were more frequent in the study group than in the control group (NS).

Figure 1 presents the means of self-efficacy at the three time points in the study and control groups. The subjects in both groups showed a high self-efficiency mean score at T_1_ (3.40—study group, 3.53—control group), which decreased at T_2_ (3.06—study group, 3.26—control group). At T_3_, the mean score of the study group rose (M = 3.34), while the mean score of the control group dropped (M = 2.95).

Table 2 shows mean scores and standard deviations of childbirth self-efficacy at T_1_–T_3_ (weighted for age). In an independent t-test analysis, there were no significant main effects for the groups at T_1_ and T_2_. A significant main effect for the groups was found at T_3_ (t(126) = 2.88, *p* < 0.01).

Repeated-measures analysis within subjects showed a significant main effect for time (F(2,246) = 12.83, *p* < 0.001, Partial Eta Squared = 8.9%).

For the interaction effect between time and group, a repeated-measures analysis within and between subjects was performed. There was a significant interaction effect between time and groups (F(2,246) = 10.20, *p* < 0.01, Partial Eta Squared = 7.2%).

Further analysis found a significant difference within the study group between the second and the third measurements, with an upward trend (t(99) = 6.24, *p* < 0.01). On the other hand, in the control group, there were significant differences in all three measurements, in a downward trend (t(28) = 2.81, *p* < 0.05).

## 4. Discussion

The current study deals with a unique population of primigravid women in the ultra-orthodox religious Jewish community of Israel. The literature showed that fear of childbirth was associated with reduced maternal confidence [1,2,3], increasing anxiety, and reduced self-efficacy [5,8]. The aim of the present study was to examine the contribution of childbirth preparation courses to a woman’s ability to cope successfully with childbirth by raising her self-efficacy.

The results in this study indicate that the preparatory course significantly contributed to self-efficacy for childbirth and allowed a higher score in the third measurement in the study group, as compared to the control group. Our findings show that regardless of the group, there were differences in the three measurements, with the first measurement results at time 1 being the highest. These results may mean that the self-efficacy of women before their first delivery was higher, corresponding to their level of expectation that they would cope with high success, than at later times. The control group had a higher self-efficacy than the intervention group. The self-efficacy of the intervention group at the end of the childbirth preparation course, and despite attending the course, was lower than that of women who did not attend the course. Another study [5] also showed a reduction in stress levels but not an increase in self-efficacy. It is possible that for this reason, women in the control group chose not to attend a childbirth preparation course. A possible explanation could be that they valued their ability to cope with childbirth successfully due to their positive assessment of their self-efficacy to cope with childbirth. As a result, they did not feel the need to attend a childbirth preparation course. According to Bandura [10,11], the source of experience as a means of strengthening self-efficacy is not the cause of high self-efficacy in childbirth, because these women have never experienced childbirth before. Therefore, they drew their sense of self-efficacy from conversations with other women about childbirth through verbal persuasion [19]. Women who attended a childbirth preparatory course may, similar to women who did not attend the course, have participated in conversations with other women who had already given birth and received support through their encouragement. Yet, they received formal and professional birth information that women who did not attend the course did not receive. Moreover, according to the theory of self-efficacy, high self-efficacy is very important because it is the one determinant that affects the motivation and the way an individual will approach and deal with a task, but knowledge and familiarity with the task is required to assess self-efficacy [19].

In the second measurement, the self-efficacy of the two groups decreased, but the difference was noticeable in the third measurement after birth, when there was a significant change, with the readiness for childbirth of women who had taken a childbirth preparation course increasing again, while among women who had not taken the course, the decline continued. Hence the gap between women’s expectation of self-efficacy at birth and the result of postpartum self-efficacy was large and negative among women who did not take a course compared to women who did take a course. Despite the decrease in self-efficacy in both groups in the second measurement, the coping of women who participated in the course was higher than that of women who did not participate in it. High self-efficacy was associated with low-risk pregnancies, fewer caesarean sections, less labor pains and suffering, and low postpartum depression rates [7], better positive assessment of the birth experience, and higher satisfaction with childbirth [8]. In agreement with the literature, in our study, women who participated in the course learned about professional coping strategies in dealing with the birth itself, while women who did not attend the course did not receive, for example, breathing exercises. As a result, the assessment of retrospective coping was negative among women who did not attend the course.

These findings have implications for women who did not attend the course. These women who approached childbirth with high self-efficacy, but apparently without sufficiently good and accurate knowledge and without birth skills or effective coping strategies acquired in the course, were left with a feeling that they did not succeed as expected. The gap between their expected and real self-efficacy before and after birth led to a negative experience. These findings raise concerns about subsequent births among these women, especially since this is a population of ultra-orthodox women, whose fertility rate is high. These women may face psychological and physical difficulties in future births. Psychologically, after the initial experience, they have a self-measure of their self-efficacy to give birth, and this may cause them fears and disappointment. As a result of this experience, according to the theories of social learning and of self-efficacy at birth [10,11], in the next birth, their self-efficacy before birth will likely be low. Low self-efficacy in childbirth correlates with fear and apprehension of childbirth leading to tocophobia, stress, difficulty in coping with labor pains, demand for cesarean section [2,5,9]. Although in the current study. the rate of caesarean sections was low (8.5%), the rate of caesarean sections is high worldwide. In Israel the proportion of caesarean section among Jewish women is 47.5% [20].

Our study has a number of strengths. First, the study group was similar to the control group, lending further validity to the findings and strengthening the importance of the course. Second, the sample size was sufficiently large for the type of analysis required. Third, the study focused on a culturally unique group, for whom the importance of childbirth is significant and which, therefore, deserves to be addressed separately from the general population. Compared to secular women, ultra-orthodox religious women have fewer options for self-learning such as the internet that provides childbirth preparation courses, social networks, and/or counseling fora, CDs, or books. Ultra-orthodox religious women’s learning opportunities about childbirth are more limited for reasons of modesty and due to limitations in the sources of learning that must be approved by religious authorities.

This study has a few limitations that should be acknowledged. First, compared with secular women, religious women have fewer options for self-learning because of their modesty and of lack of access to resources of learning, which limits the generalizability of these results to the entire population of Israeli women. In addition, it does not represent women who decided to give birth outside a hospital. Second, the only significant difference between the study and the control groups was the age of the participants (3 years older in the control group than in the study group). The participants were not randomly selected into the groups for ethical reasons, and it might be that the younger felt less prepared, while the older believed to a lesser extent they needed this course, as they felt more mature and had had more chances to listen to friends’ experiences or read and have advice compared to the youngest. These factors might have affected the results of the study. Third, the results of this study show that the control group had a higher frequency of ‘natural’ births and a lower frequency of obstetric procedures than the intervention group, though not significantly so. These differences, if were significant, would be opposed to the suggested hypothesis and raise the question of whether self-efficacy scores decreased immediately after birth because these women felt they had failed to implement the self-help strategies they had been taught. This point should be addressed in a future study.

## 5. Conclusions

In contrast to some literature on childbirth preparation courses, this study presents evidence that these courses benefit primigravid women in the religious sector. The popularity of these courses has declined because of the possibility of learning through the internet and the rise in caesarean sections. However, participation in them can be recommended, at least in the population of religious women. In this population, participating in a course raised women’s self-efficacy to cope successfully in childbirth. 

The practical recommendation following our findings is to continue to hold childbirth preparation courses and to encourage women who are undecided whether to attend the course to make a positive decision. It is desirable that future research includes groups of secular Jewish and Arab women, as well as other ethnic groups, in order to allow a more sweeping generalization for primigravid women in the Israeli society. For some ethnic groups, in which the pregnancy age of the majority is low compared to that of the average population, it is especially advisable to offer these courses in native languages and in a culturally adapted way. Alternatively, it will be important to compare birth preparation courses according to different teaching methods, such as online learning.

## Figures and Tables

**Table 1:** Socio-demographic characteristics of the study and control groups (unweighted sample).

Variable		Study Group *n* = 100	Control Group *n* = 30	Sig.	Total *n* = 130
Intervention	Yes/no	76.9% yes	23.1% no	-	100%
Subject’s age	Mean (SD)	23.4 (4.3)	26.1 (6.1)	**0.000**	24.0 (4.9)
Family status	Married	100%	96.6%	N.S.	99.2%
	Divorced	0.0%	3.4%	N.S.	0.8%
Country birth	Israel	93.0%	96.6%	N.S.	93.8%
	Other	7.0%	3.4%	N.S.	6.2%
Education	Non-academic	49.5%	50.5%	N.S.	49.6%
	Academic/professional	50.5%	50.5%	N.S.	50.4%
Pregnant age	Mean (SD)	32.9 (4.2)	32.8 (5.3)	N.S.	32.9 (4.4)
Birth week	Mean (SD)	39.6 (1.3)	39.6 (1.3)	N.S.	39.6 (1.3)
Birth method	Natural	76.8%	86.7%	N.S.	79.1%
	Caesarean	9.1%	6.7%	N.S.	8.5%
	Instrumental	14.1%	6.7%	N.S.	12.4%
Healthy pregnancy	Yes	89.0%	83.3%	N.S.	87.7%
	No	11.0%	16.7%	N.S.	12.3%
Pain relief such as epidural	Yes	91.0%	83.3%	N.S.	89.2%
	No	9.0%	16.7%	N.S.	10.8%
Induction of birth	Yes	44.4%	43.3%	N.S.	44.2%
	No	55.6%	56.7%	N.S.	55.8%
Nursing	Yes	94.9%	90.0%	N.S.	93.8%
	No	5.1%	10.0%	N.S.	6.2%

**Table 2:** Mean scores and standard deviations of childbirth self-efficacy at T_1_–T_3_ (weighted for age).

	Study Group *n* = 99		Control Group *n* = 28		Total *n* = 127 *		
	Mean	SD	Mean	SD	Mean	SD	*p*-Values for Group
T_1_	3.40	0.63	3.53	0.56	3.44	0.63	*p* > 0.05
T_2_	3.06	0.76	3.26	0.63	3.14	0.75	*p* > 0.05
T_3_	3.34	0.64	2.95	0.76	3.23	0.69	t(126) = 2.88, *p* < 0.01
	*p*-values for time, F(2,246) = 12.83, *p* < 0.001						

* Three participants were dropped from the analysis due to missing values.

## Data Availability

Data available on request due to privacy restrictions.
